# A Two-Level Illumination Correction Network for Digital Meter Reading Recognition in Non-Uniform Low-Light Conditions

**DOI:** 10.3390/jimaging12040146

**Published:** 2026-03-25

**Authors:** Haoning Fu, Zhiwei Xie, Wenzhu Jiang, Xingjiang Ma, Dongying Yang

**Affiliations:** 1 College of Computer Science and Cybersecurity, Chengdu University of Technology, Chengdu 610059, China; 202318010406@stu.cdut.edu.cn; 2 State Key Laboratory of Public Big Data, Guizhou University, Guiyang 550025, China

**Keywords:** digital meter reading, autonomous recognition, non-uniform low-light conditions, deep learning, YOLOv10

## Abstract

The automatic reading recognition of digital instruments is crucial for achieving metering automation and intelligent inspection. However, in non-standardized industrial environments, the masking effect caused by the coupling of non-uniform low-light conditions and the reflective surfaces of instrument panels severely degrades the displayed information, significantly limiting the recognition performance. Conventional image processing methods, while aiming to restore the imaging quality of instrument panels through low-light enhancement, inevitably introduce overexposure and indiscriminately amplify background noise during this process. To address the two key challenges of illumination recovery and noise suppression in the process of restoring panel image quality under non-uniform low-light conditions, this paper proposes a coarse-to-fine cascaded perception framework (CFCP). First, a lightweight YOLOv10 detector is employed to coarsely localize the meter reading region under non-uniform illumination conditions. Second, an Adaptive Illumination Correction Module (AICM) is designed to decouple and correct the illumination component at the pixel level, effectively restoring details in dark areas. Then, an Illumination-invariant Feature Perception Module (IFPM) is embedded at the feature level to dynamically perceive illumination-invariant features and filter out noise interference. Finally, the refined detection results are fed into a lightweight sequence recognition network to obtain the final meter readings. Experiments on a self-built industrial digital instrument dataset show that the proposed method achieves 93.2% recognition accuracy, with 17.1 ms latency and only 7.9 M parameters.

## 1. Introduction

Metrology, as the science of measurement, focuses on ensuring the accuracy, consistency, and traceability of measurement value transmission. It provides foundational support for critical industrial sectors, such as petrochemicals and power grids, which demand exceptionally high levels of safety and stability [1]. However, the reliable acquisition of instrument readings, a crucial step in achieving metrological goals, still predominantly relies on manual inspection. This method is labor-intensive, inefficient, and prone to human error [2,3]. Therefore, developing an automatic Digital Meter Reading Recognition (DMRR) method that meets the high-precision and high-reliability requirements of metrology is not only an urgent need for industrial intelligent transformation but also a key enabling technology for enhancing the automation level of industrial measurement and ensuring the integrity of value transmission.

Early research on DMRR primarily focused on traditional digital image processing techniques, such as segmentation based on color features, grayscale projection methods, and template matching [4,5]. These methods generally rely on manually designed heuristic rules and feature operators to achieve localization of the reading region and character segmentation. Although satisfactory performance can be achieved in controlled laboratory environments, their core limitation lies in a lack of inherent generalization capability against complex environmental disturbances. In real-world industrial scenarios, common factors such as subtle lighting fluctuations, stains on the instrument panel, or local shadow occlusion can easily lead to failures in binarization thresholding or incomplete feature extraction [6,7,8]. Consequently, such methods relying on fixed priors struggle to satisfy the stringent requirements of industrial sites for all-weather and highly reliable monitoring. This limitation has led researchers to turn to deep learning-based approaches. However, even though deep learning compensates for the shortcomings of traditional methods, how to correct illumination while avoiding the amplification of background noise in real non-uniform low-light environments remains a critical challenge that needs to be addressed.

**Figure 1:**
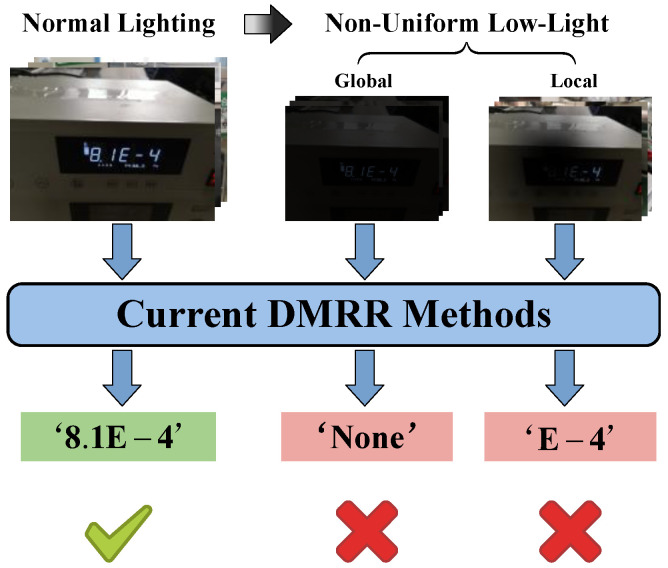
Performance degradation of existing DMRR methods in non-uniform low-light environments. Existing DMRR methods often fail to detect readings or produce incorrect readings under non-uniform low-light conditions.

Existing deep learning-based methods have effectively overcome the reliance of traditional approaches on manual features and fixed parameters. With their powerful generalization capabilities in complex scenarios, they have become the mainstream paradigm in the field of DMRR [9]. Most current research follows a two-stage framework of detection followed by recognition. Specifically, generic detectors such as the YOLO [10,11,12] or R-CNN [13,14,15] series are first employed to localize the instrument area. Subsequently, sequence recognition networks like CRNN [16] or DBNet [17,18] are used to convert the localized region into a digital sequence. To pursue higher efficiency, some studies have attempted end-to-end solutions. For instance, the Fast-OCR framework proposed by Laroca et al. [19] demonstrates excellent performance under conventional lighting conditions. However, both two-stage methods and end-to-end methods experience a significant performance degradation in real-world non-uniform low-light industrial environments (as shown in Figure 1). The core issue lies in the fact that existing approaches typically treat low-light enhancement as an isolated pre-processing problem, directly applying general-purpose image enhancement modules such as RetinexNet or Zero-DCE for global brightness improvement. This simplistic image-level enhancement strategy suffers from an inherent flaw: while boosting brightness, it indiscriminately amplifies interfering background noise, such as stains and scratches on the instrument panel, thereby overwhelming the digital semantic features that are crucial for recognition. Consequently, the synergistic performance of illumination correction and noise suppression has become a critical challenge to be addressed for achieving high-precision and robust DMRR. This task specifically requires the restoration of reading blind spots while simultaneously protecting and enhancing digital semantic features vital for recognition, rather than merely improving visual visibility.

To achieve effective noise suppression while performing illumination recovery, this paper proposes a coarse-to-fine cascaded perception framework (CFCP). Its innovation lies in integrating a Two-Level Illumination Correction Network (TLICN) during the detection stage, which directly addresses the coupled interference of low-light conditions and noise through a synergistic mechanism of pixel-level correction and feature-level perception. CFCP follows the two-stage DMRR paradigm of detection followed by recognition. In the first stage, a lightweight YOLOv10 [20] detector is first employed for coarse localization and scale normalization of the instrument region. Subsequently, the TLICN performs pixel-level illumination correction on the reading area image using its Adaptive Illumination Correction Module (AICM), which is based on Retinex theory [21]. This process eliminates reading blind spots and restores key areas obscured by low-light conditions without introducing additional artifacts. Subsequently, the Illumination-invariant Feature Perception Module (IFPM) within TLICN operates in the feature-level, utilizing multi-scale attention to dynamically suppress background noise amplified by the illumination correction and guide TLICN to focus on digital semantic features. This facilitates the synergistic processing of illumination restoration and noise suppression. In the second stage, the target reading region, after illumination correction and fine segmentation, is fed into a lightweight sequence recognition network to output the final accurate meter reading. From coarse regional localization, physical illumination decoupling, and illumination-invariant feature extraction to reading recognition, all customized components form a complete synergistic pipeline. Experiments on a self-built non-uniform low-light digital instrument dataset demonstrate that the proposed method achieves a balance between recognition accuracy and inference speed, outperforming existing mainstream approaches. In summary, the main contribution of this study are as follows:

- We design an Adaptive Illumination Correction Module (AICM) based on Retinex theory to address the issue of reading blind spots caused by non-uniform illumination. Different from conventional image enhancement methods, this module adaptively decouples the illumination and reflectance components, effectively eliminating visual blind spots without introducing artifacts, thereby achieving pixel-level illumination correction for the input data.
- To address the inevitable amplification of background noise during illumination enhancement, we construct an Illumination-invariant Feature Perception Module (IFPM). Leveraging channel attention and spatial attention as feature filters, IFPM dynamically suppresses non-semantic background noise and guides the network to focus on digital semantic features, thereby achieving the synergy between illumination recovery and illumination-invariant feature extraction.
- Building upon these customized modules, we propose a coarse-to-fine cascaded perception framework (CFCP) for DMRR in non-uniform low-light environments. This framework deeply integrates the coarse localization of YOLOv10 with fine-grained text segmentation and recognition. By employing a multi-stage processing strategy, it achieves efficient noise suppression while simultaneously performing illumination recovery.

## 2. Related Work

The Digital Meter Reading Recognition (DMRR) task aims to identify the readings in instrument images with complex backgrounds as digital sequences. Reviewing the development of this field, the research focus has shifted from early recognition in ideal environments to adapting to more challenging real-world industrial scenarios. Furthermore, given the inevitably harsh lighting conditions at industrial sites, Low-Light Image Enhancement (LLIE) techniques also serve as a critical auxiliary means for addressing such challenges. This section will systematically review related work in DMRR methods and low-light enhancement technologies. By analyzing the challenges faced by existing studies under non-uniform low-light conditions, it reveals the research motivation for the proposed approach.

### 2.1. Digital Meter Reading Recognition Methods

Early research on DMRR primarily relied on traditional digital image processing techniques and heuristic rules. For example, Anis et al. [22] utilized connected component filtering and morphological processing for character segmentation, while Bai et al. [23] employed projection-based methods for reading localization. However, such methods heavily depend on manually designed feature operators and are highly sensitive to environmental conditions. Their generalization capability is limited in non-standardized industrial scenarios, often leading to localization failures or character segmentation errors. With the advancement of deep learning, progress in object detection algorithms has significantly driven the development of this field. Some researchers have begun to explore high-performance object detectors to address the challenge of reading localization. For instance, Shuo et al. [24] employed MobileNet-SSD for lightweight localization, while Iqbal et al. [25] utilized a single-stage detector based on the Inception architecture. Such localization methods are lightweight and achieve accurate positioning for horizontally captured meters. However, maintaining a horizontal shooting angle is often difficult in industrial settings. To address the perspective distortion caused by shooting angles, Zhang et al. [26] introduced a Stacked Hourglass Network (SHN) for keypoint localization, and Laroca et al. [19] combined Fast-YOLO [27] with CDCC-Net to achieve corner detection and geometric correction. These point-based localization methods exhibit strong robustness to varying shooting angles. After completing the detection of the reading region, the cropped samples are typically fed into sequence recognition networks such as CRNN to extract the digital sequence and obtain the final reading result. To further simplify the above detection-recognition pipeline, some researchers have attempted to integrate detection and recognition into a unified process. For example, Gómez et al. [28] proposed a segmentation-free system capable of directly regressing the digital sequence without pre-localizing the text region. Meanwhile, the method based on YOLOv3 [29] by Imran et al. [2] innovatively transformed character recognition into an object detection problem, thereby achieving integrated localization and character recognition. Furthermore, Li et al. [30] introduced an attention mechanism on this basis to enhance the network’s ability to focus on relevant features in complex backgrounds, thereby improving the final accuracy of digital category recognition.

Despite the significant progress made by the aforementioned deep learning-based DMRR methods in reading localization and integrated recognition, they still face substantial challenges when confronted with non-uniform low-light conditions in real industrial scenarios. Two-stage detection-recognition models suffer from considerable errors in reading region detection at the detection stage due to the adverse effects of poor illumination, which subsequently propagates errors to the recognition stage. Integrated models, while focused on extracting holistic reading features, often find key information obscured by non-uniform low-light conditions, leading to ineffective feature extraction and, consequently, compromised reading accuracy. Therefore, the key to addressing this issue lies in fundamentally restoring the reading blind spots at the pixel level before feature extraction. In CFCP, we introduce the AICM, based on the physical prior of Retinex theory, during the detection stage. By adaptively decoupling illumination and reflectance components, it effectively eliminates reading blind spots.

### 2.2. Low-Light Image Enhancement

Low-Light Image Enhancement (LLIE) aims to restore image details under poor illumination conditions. Existing enhancement methods are broadly categorized into traditional methods and machine learning-based methods. Traditional methods primarily rely on Histogram Equalization (HE) or Retinex theory [21]. Among these, methods based on Retinex theory assume that the reflectance component remains consistent under varying illumination. They decompose an image into an illumination map and a reflectance map for enhancement. For instance, Fu et al. [31,32] utilized norm constraints to propose variational optimization-based solutions, while the Retinex model proposed by Li et al. [33] further incorporated a noise term, estimating the illumination distribution by solving an optimization problem. However, these methods typically rely on handcrafted prior features and struggle to simultaneously achieve denoising and detail restoration under complex lighting conditions. In recent years, deep learning-based approaches have gradually become mainstream, primarily comprising supervised and unsupervised learning methods. Supervised methods, such as LLNet [34] and RetinexNet [35], have made significant progress. Notably, RetinexNet innovatively integrated Retinex decomposition theory with convolutional neural networks. However, such methods heavily rely on paired training data, which is often difficult to acquire. To address the scarcity of data, Zero-DCE [36] models enhancement as a task of estimating specific image curves through unsupervised learning. EnlightenGAN [37] introduced generative adversarial networks to enable training with unpaired data, while Cui et al. [38] proposed the Transformer-based IAT model to adaptively adjust ISP parameters.

Although the aforementioned illumination enhancement algorithms excel in improving visual brightness and contrast, they are primarily designed for human visual perception. In the context of industrial digital instrument scenarios, directly applying these general-purpose algorithms often amplifies background noise (such as screen dust and scratches) while brightening the image. This amplification of noise severely degrades the edge semantic features of digital characters, leading to a decline in the accuracy of downstream recognition. Therefore, after performing illumination enhancement on instrument images, suppressing the amplified noise is crucial for ensuring the reliability of the final reading. To address this issue, we constructed the IFPM, which employs a multi-scale attention mechanism as a feature filter to dynamically suppress non-semantic background noise and guide the network to focus on digital semantic features. By working in synergy with the AICM, it successfully achieves both the elimination of reading blind spots and the suppression of amplified background noise.

## 3. Methodology

### 3.1. Overview of the CFCP Framework

**Figure 2:**
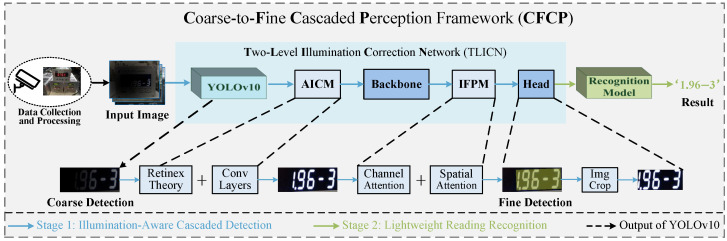
Overall workflow and architecture of the proposed Coarse-to-Fine Cascaded Perception Framework (CFCP). The framework comprises Illumination-Aware Cascaded Detection (Stage 1) and Reading Recognition (Stage 2). By integrating the YOLOv10 coarse detector, the pixel-level AICM module, and the feature-level IFPM module, CFCP effectively overcomes non-uniform low-light interference and suppresses noise amplified by illumination enhancement, achieving coarse-to-fine detection of digital instrument reading regions and accurate reading transcription. The Fine Detection example image in the figure is the final output of TLICN, where the yellow shaded area indicates the finely detected reading region, which is subsequently segmented to serve as the input to the recognition model.

As shown in Figure 2, we collected samples using industrial cameras in a real-world meter inspection laboratory. After data preprocessing and augmentation, the resulting input samples are fed into the proposed Coarse-to-Fine Cascaded Perception Framework (CFCP) for detection and recognition. CFCP is designed to structurally decouple the physical perception of illumination and the semantic understanding of digital features in non-uniform low-light industrial environments through a hierarchical processing strategy. Furthermore, the proposed framework supports the detection and recognition of multi-line readings. During the detection stage, the framework outputs independent instance-level bounding boxes for each line of text, and the recognition network subsequently performs recognition on these cropped reading regions individually. The two core stages of CFCP are as follows:

Stage 1: Illumination-Aware Cascaded Detection. The first stage of CFCP is designed as a Two-Level Illumination Correction Network (TLICN), aimed at accurately locating and restoring reading blind spots in digital instruments under non-uniform low-light conditions. TLICN adopts a coarse-to-fine processing strategy. The process begins with coarse detection and background filtering. Considering the complex backgrounds typical of industrial scenes, a lightweight general purpose object detector (YOLOv10 in this study) is first employed as the coarse detection network for reading regions. This initial step focuses not on precise segmentation, but on rapidly filtering out most non-instrument background interference and generating preliminary regions of interest (RoI) containing the instrument panel. The coarse detection results serve as the foundational input for subsequent refinement. This is followed by illumination recovery and fine detection based on AICM and IFPM. The RoI obtained from coarse localization are processed by the AICM, which performs pixel-level illumination component decoupling based on Retinex theory to eliminate visual blind spots. Subsequently, the features extracted by the backbone network are directed to the IFPM. This module utilizes a multi-scale attention mechanism to dynamically suppress background noise amplified during illumination enhancement at the feature level. Finally, these semantically enhanced features, optimized at both pixel and feature levels, are delivered to the detection head for regression of the final precise reading bounding boxes.

Stage 2: Lightweight Reading Recognition. After obtaining precise character localization (as indicated by the green flow in Figure 2), the character regions are cropped and rectified according to the bounding boxes. These standardized character images are then fed into the reading recognition network, where visual features are decoded into the final digital readings. The subsequent sections of this chapter will elaborate in detail on the design specifics of the aforementioned core modules.

### 3.2. Illumination-Aware Cascaded Detection

#### 3.2.1. Coarse Detection

In DMRR tasks, the originally captured images often possess high resolution and are cluttered with complex backgrounds such as wiring, walls, and operators. This distribution characteristic of large backgrounds and small targets can significantly diminish the feature response of digital characters, making direct recognition extremely challenging. Therefore, as the first step in CFCP, an efficient mechanism is required to quickly locate and roughly isolate the instrument region, thereby eliminating background interference for subsequent fine-grained processing.

We have selected YOLOv10 [20] as the coarse detector. Compared to the widely deployed YOLOv8 [11] and the architecturally optimized YOLOv9 [39], YOLOv10 introduces a Consistent Dual Assignments strategy, completely eliminating reliance on Non-Maximum Suppression (NMS). This fundamentally removes the inference latency bottleneck during post-processing that is typical of traditional YOLO series models. Benchmark results reported in the original paper demonstrate that, while maintaining comparable accuracy, YOLOv10 achieves significant reductions in both parameter count and latency compared to YOLOv8. This provides the optimal efficiency-accuracy tradeoff, satisfying the stringent real-time requirements of industrial edge deployment [20].

#### 3.2.2. Adaptive Illumination Correction Module

**Figure 3:**
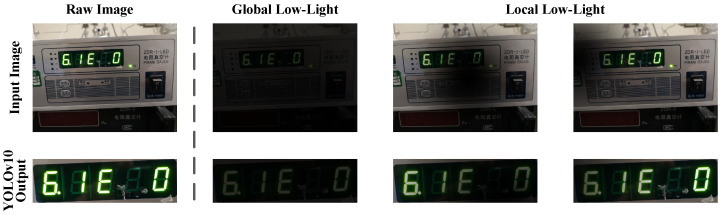
Motivation analysis of the cascaded architecture design. Even under global low-light or local shadow interference, YOLOv10 can still accurately coarsely localize the instrument panel region.

Although the coarse detection stage achieves spatial normalization of the digital instrument RoI, the cropped RoI image $I_{roi}$ remains affected by non-uniform low-light conditions. This physical-level loss of visibility leads to visual blind spots for the feature extractor. Before introducing the Adaptive Illumination Correction Module (AICM), we justify the rationale for deploying it after YOLOv10. As shown in Figure 3, we observe that the coarse-level panel structure features extracted by YOLOv10 demonstrate relatively good robustness to illumination variations. They can still detect the instrument region even under global low-light or local shadow interference. If a full-image pre-enhancement strategy were adopted, it would not only introduce an additional computational overhead of O(H×W) but could also amplify noise in background areas. Therefore, we choose to introduce the AICM after RoI cropping. This strategy reduces the computational complexity from the megapixel scale to the tens-of-kilopixel scale, thereby improving the overall efficiency of CFCP to some extent.

**Figure 4:**
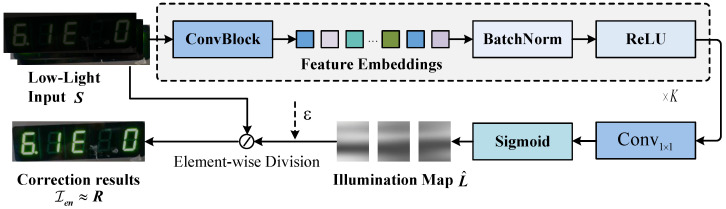
Schematic diagram of the AICM structure. Based on Retinex theory, the illumination map is extracted through a *K*-level cascaded fully convolutional network. After obtaining the illumination map $\hat{L}$, the illumination restoration process is formulated as a physical inverse operation, and a regularization term ε is introduced to avoid division by zero. The final image after illumination restoration is obtained.

As shown in Figure 4, the AICM is constructed based on Retinex theory. For any single low-light image *S* within the input tensor $I_{roi}$, its physical imaging process can be modeled as the element-wise coupling of the scene reflectance *R* and the ambient illumination field *L*:

(1) $$\begin{array}{c} S(x,y)=R(x,y)⊙L(x,y) \end{array}$$

where (x,y) denotes pixel coordinates, and ⊙ represents the Hadamard product. In this model, *R* characterizes the intrinsic physical properties of the object surface (i.e., the digital character features), while *L* represents the non-uniform illumination distribution causing image degradation. Traditional Retinex methods [21] typically rely on handcrafted Gaussian filters or simplistic smoothing assumptions to solve for *R* and *L*, which are prone to generating halo artifacts or color distortion around sharp edges. In contrast, when estimating the illumination component, the AICM leverages the spatial smoothness prior of the illumination field [40] and formulates the illumination estimation as a deep convolutional regression process driven by parameters Θ. Specifically, the AICM is instantiated as a fully convolutional network comprising *K* cascaded layers. Given the input *S*, the feature extraction process at the *k*-th layer is defined as:

(2) $$\begin{array}{c} F_{k}=\delta \left(BN\left(\mathrm{Con}v_{k}\left(F_{k-1};W_{k},b_{k}\right)\right)\right),\;\;\;k=1,\ldots ,K \end{array}$$

where $F_{0}=S$ represents the input image. ${\mathrm{Conv}}_{k}\left(\cdot \right)$ denotes a convolutional operation with learnable weights $W_{k}$ and bias $b_{k}$, BN(·) represents batch normalization, and δ(·) is the ReLU activation function. By stacking multiple convolutional blocks, the network progressively enlarges its receptive field to capture the global illumination distribution. At the final convolutional stage, to obtain the estimated illumination map $\hat{L}$ and constrain its values within the interval (0,1), we employ a 1×1 convolutional layer followed by the Sigmoid activation function σ(·) for mapping:

(3) $$\begin{array}{c} \hat{L}=\sigma \left(\mathrm{Con}v_{out}\left(F_{K}\right)\right) \end{array}$$

Building upon the characteristic that ambient illumination typically exhibits spatially piecewise smooth properties, we design an Illumination Smoothness Loss $L_{smooth}$ to guide the network’s optimization direction. This loss function encourages structural consistency in $\hat{L}$ and suppresses the loss of character details by minimizing the $L_{1}$ norm of the illumination map’s gradients:

(4) $$\begin{array}{c} L_{smooth}=\sum_{x,y}\left(|\nabla_{x}\hat{L}\left(x,y\right)|+|\nabla_{y}\hat{L}\left(x,y\right)|\right) \end{array}$$

where $\nabla_{x}$ and $\nabla_{y}$ denote the gradient operators in the horizontal and vertical directions, respectively. By minimizing $L_{smooth}$, the AICM is guided to decouple character edges from low-frequency illumination variations. This constraint prevents the illumination map $\hat{L}$ from overfitting and ensures that the semantic details in the digital region are fully preserved in the final reflectance component, thereby avoiding character blurring or loss caused by the enhancement process.

After the AICM extracts the estimated illumination map $\hat{L}$, we define the reflectance recovery process as the corresponding physical inverse operation. However, a direct division operation can lead to severe numerical instability in extremely low-light regions (where $\hat{L}\to 0$) and is highly prone to introducing artifacts in very dark areas. To address this, we introduce a regularization term ε into the inverse solving process to avoid division by zero:

(5) $$\begin{array}{c} I_{en}=\hat{R}=S⊘\left(\hat{L}+\varepsilon \right) \end{array}$$

where $I_{en}$ is the enhanced image tensor recovered by AICM, and ⊘ denotes element-wise division. AICM is not merely a simple enhancer but a learnable normalization operator based on physical constraints. Before the samples enter the feature space, it explicitly decouples and corrects the ambient illumination interference, ensuring that the subsequent backbone network receives the reflectance component *R*, which reflects the intrinsic properties of the object.

#### 3.2.3. Illumination-Invariant Feature Extraction and Fusion

Taking the illumination-corrected image $I_{en}$ as input, this stage aims to extract features of the reading region that are robust to illumination variations, and to construct a feature pyramid that integrates multi-level feature representations. Although AICM effectively restores the overall visibility of the image, in extremely low-light areas, the reflectance recovery process inevitably amplifies noise present in the background (such as panel stains). The background noise introduced by this enhancement process often manifests as isolated high-response points at the feature level, which are difficult to distinguish from genuine digital stroke features through simple global statistics. To address the issue of background noise amplification accompanying the illumination enhancement process, we propose an Illumination-invariant Feature Perception Module (IFPM). Considering the requirements for edge deployment and lightweight design, this module employs MobileNetV3 [41] as its feature extraction backbone. Moreover, the SE module within the original MobileNetV3 relies solely on Global Average Pooling (GAP). This global statistical operation tends to smooth feature distributions, making it highly susceptible to submerging subtle digital stroke features in the amplified background noise. To address this, we deploy IFPM at four key feature stages in MobileNetV3 (i.e., $F_{1},\ldots ,F_{4}$). IFPM introduces a parallel max-pooling branch to preserve character texture features, thereby suppressing isolated noise responses while precisely focusing on the geometric structure of digits.

**Figure 5:**
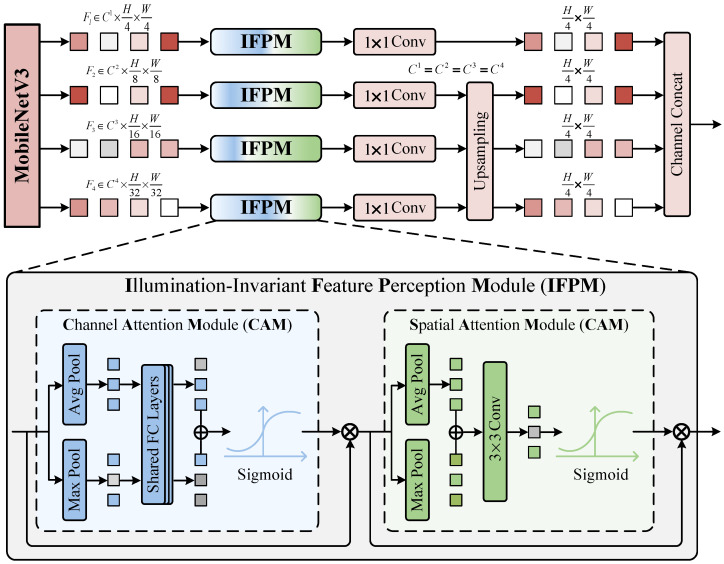
Overview of the Illumination-invariant Feature Perception Module (IFPM) and its collaborative strategy with the MobileNetV3 backbone. IFPM operates on the output features $F_{1}\ldots F_{4}$ at four different scales. The Channel Attention Module (CAM) adaptively recalibrates channel weights using parallel max-pooling and average-pooling operations; the Spatial Attention Module (SAM) precisely locates the reading region through pooling layers and a 3×3 convolution.

The structure of IFPM is illustrated in Figure 5. Its inputs are the feature tensors $F_{i}\in R^{B\times C\times H\times W}$ output by MobileNetV3 at four stages, where *B* denotes the batch size. This module calibrates the features by sequentially applying channel attention (CAM) and spatial attention (SAM). To compensate for the loss of detail inherent in the native SE module, CAM introduces a dual-pooling strategy. Global average pooling and global max pooling are performed in parallel on the input feature $F_{i}$. Average pooling tends to aggregate overall contextual information, thereby sensing the background illumination distribution, while max pooling focuses on extracting salient digital stroke features, preserving the characteristics of digit edges. The resulting statistical vectors are mapped by a shared multi-layer perceptron (Shared MLP) and then concatenated along the channel dimension to obtain the aggregated feature $F_{agg}$:

(6) $$\begin{array}{c} F_{agg}=\mathrm{MLP}\left(\mathrm{AvgPool}\left(F_{i}\right)\right)⊕\mathrm{MLP}\left(\mathrm{MaxPool}\left(F_{i}\right)\right) \end{array}$$

Subsequently, the channel weights $M_{c}\in R^{B\times C\times 1\times 1}$ are generated via the Sigmoid activation function, and the input features are weighted and calibrated accordingly:

(7) $$\begin{array}{c} M_{c}\left(F_{i}\right)=\sigma \left(F_{agg}\right) \end{array}$$

(8) $$\begin{array}{c} {F^{\prime }}_{i}=M_{c}\left(F_{i}\right)⊗F_{i} \end{array}$$

SAM is designed to utilize local spatial context to distinguish continuous digital strokes from isolated background noise. First, intermediate features $F_{i}^{\prime }$, processed by CAM, undergo average pooling and max pooling separately along the channel dimension. The resulting two two-dimensional features are then concatenated along the channel dimension to obtain the global spatial feature context $F_{s}$:

(9) $$\begin{array}{c} F_{s}=\mathrm{Concat}\left(\mathrm{AvgPool}\left({F^{\prime }}_{i}\right);\mathrm{MaxPool}\left({F^{\prime }}_{i}\right)\right) \end{array}$$

where $F_{s}\in R^{B\times 2\times H\times W}$. A 3×3 convolutional layer is then employed to aggregate local neighborhood information, generating the spatial mask $M_{s}\in R^{B\times 1\times H\times W}$, which subsequently yields the final illumination-invariant features $F_{i}^{inv}$:

(10) $$\begin{array}{c} M_{s}\left({F^{\prime }}_{i}\right)=\sigma \left(\mathrm{Con}v^{3\times 3}\left(F_{s}\right)\right) \end{array}$$

(11) $$\begin{array}{c} F_{i}^{inv}=M_{s}\left({F^{\prime }}_{i}\right)⊗{F^{\prime }}_{i} \end{array}$$

In this process, the local receptive field of the 3×3 convolutional kernel plays a crucial role. It effectively identifies spatially continuous digital stroke features while suppressing background noise features that are distributed in isolated patterns.

After layer-wise calibration by the IFPM, we obtain a set of illumination-invariant features $F_{1}^{inv},F_{2}^{inv},F_{3}^{inv},F_{4}^{inv}$. However, these features are distributed across different spatial resolutions. While deep-layer features contain the topological structural information of the digits, their spatial resolution is too low to meet the segmentation accuracy requirements of the subsequent detection head. To address this, we construct a feature pyramid network (as shown in Figure 5). It is designed to map the deep structural features back to the high-resolution spatial domain and perform a physical concatenation with the shallow features. Specifically, we first use 1×1 convolutions to project the channel numbers of all feature maps uniformly to $C_{out}=256$, thereby eliminating discrepancies in the channel dimension. Subsequently, following a bottom-up path, the deep features $F_{2}^{inv},F_{3}^{inv},F_{4}^{inv}$ are progressively upsampled via bilinear interpolation to match the spatial resolution of the shallowest feature $F_{1}^{inv}$ (i.e., 1/4 of the original image size). Finally, the aligned four feature groups are concatenated along the channel dimension to generate the final fused feature $P_{fuse}$:

(12) $$\begin{array}{c} P_{fuse}=\mathrm{Concat}\left(F_{1}^{inv},U\left(F_{2}^{inv}\right),U\left(F_{3}^{inv}\right),U\left(F_{4}^{inv}\right)\right) \end{array}$$

where U(·) denotes the upsampling operation. Through this process, $P_{fuse}$ successfully aggregates key features distributed across different scales into a single high-resolution dense feature map. It not only retains the discriminative power of deep networks for identifying digit regions, but also restores the spatial localization of character edges from shallow networks. Consequently, it provides a comprehensive feature representation for the subsequent binary segmentation prediction.

#### 3.2.4. Prediction Head and Joint Optimization Objective

Inspired by DBNet [17,18], we adopt a Differentiable Binarization (DB) strategy to generate the final segmentation result and achieve end-to-end joint training through a combined loss function. To map the multi-scale fused feature $P_{fuse}$ into a geometrically meaningful prediction map, we construct parallel convolutional branches at the end of the feature pyramid. Specifically, $P_{fuse}$ first undergoes further contextual encoding via a 3×3 convolutional layer, then proceeds to two independent processing branches. The probability prediction branch outputs a probability map $P\in R^{H\times W}$, where $P_{i,j}\in \left(0,1\right)$ represents the confidence that pixel (i,j) belongs to the digit stroke foreground. This is achieved by using a 1×1 convolution to reduce the channel dimension to 1, followed by the Sigmoid activation function σ to map the feature values into the interval (0,1):

(13) $$\begin{array}{c} P=\sigma ({\mathrm{Conv}}_{1\times 1}\left(P_{fuse}\right)) \end{array}$$

Similarly, the threshold prediction branch outputs a threshold map $T\in R^{H\times W}$, which predicts an adaptive binarization threshold for each pixel to accommodate local contrast variations in different regions:

(14) $$\begin{array}{c} T=\sigma ({\mathrm{Conv}}_{1\times 1}\left(P_{fuse}\right)) \end{array}$$

To make the binarization process differentiable during training, DBNet replaces the standard step function with a smooth approximation function to dynamically generate the approximate binary map $\hat{B}$:

(15) $$\begin{array}{c} {\hat{B}}_{i,j}=\frac{1}{1+e^{-k(P_{i,j}-T_{i,j})}} \end{array}$$

where the scaling factor *k* is empirically set to 50. This formulation approximates the standard binarization function while exhibiting a steep gradient near the decision boundary. Consequently, the network can adaptively and jointly optimize the probability map *P* and the threshold map *T* based on the feedback from the segmentation results.

To jointly optimize illumination enhancement effectiveness and text segmentation accuracy within a single forward pass, we define the total loss function $L_{total}$ as a weighted sum of the detection task loss $L_{det}$ and the illumination physical constraint loss $L_{smooth}$:

(16) $$\begin{array}{c} L_{total}=L_{det}+\lambda L_{smooth} \end{array}$$

where λ is a hyperparameter used to balance the weights of the two tasks (set to 0.3 in our experiments), controlling the influence strength of the illumination constraint on the feature extraction network. The detection loss $L_{det}$ comprises the probability map loss $L_{s}$, the binary map loss $L_{b}$, and the threshold map loss $L_{t}$:

(17) $$\begin{array}{c} L_{det}=L_{s}+\alpha L_{b}+\beta L_{t} \end{array}$$

where α and β are set to 1.0 and 10.0, respectively. Following the Online Hard Example Mining (OHEM) strategy [42], to address the severe sample imbalance caused by the extremely low proportion of foreground stroke pixels in the instrument panel, we denote all positive samples (digit stroke regions) as the set $S_{pos}$. For negative samples (background regions), we calculate the loss values of all background pixels, sort them in descending order, and select the top $N_{neg}$ pixels with the highest loss values to form the hard negative sample set $S_{neg}$, maintaining the ratio $|S_{neg}\left|\;:\;\right|S_{pos}|=3:1$. Let $S_{l}=S_{pos}\cup S_{neg}$ be the final sampled set participating in training. The probability map loss $L_{s}$ is defined using Binary Cross-Entropy (BCE) as:

(18) $$\begin{array}{c} L_{s}=\frac{1}{|S_{l}|}\sum_{\left(i,j\right)\in S_{l}}-\left(G_{i,j}\log P_{i,j}+\left(1-G_{i,j}\right)\log\left(1-P_{i,j}\right)\right) \end{array}$$

where $G_{i,j}\in \left\{0,1\right\}$ is the ground truth label for that pixel. The binary map loss $L_{b}$ aims to supervise the generation quality of the approximate binary map $\hat{B}$ and is also computed as a BCE loss on the OHEM sampled set $S_{l}$:

(19) $$\begin{array}{c} L_{b}=\frac{1}{|S_{l}|}\sum_{\left(i,j\right)\in S_{l}}-\left(G_{i,j}\log{\hat{B}}_{i,j}+\left(1-G_{i,j}\right)\log\left(1-{\hat{B}}_{i,j}\right)\right) \end{array}$$

This term directly constrains and guides the binarized result towards the ground truth, ensuring the network learns the correct binarization boundaries. Secondly, the threshold map loss $L_{t}$ is employed to guide the network in learning the morphology of the character boundaries. The threshold map focuses on the transitional regions near the reading boundaries. We expand the original polygon outward to obtain an expanded polygon $G_{d}$. Subsequently, the annular region between $G_{d}$ and the shrunk region $G_{s}$, namely the reading boundary, is defined as the effective supervision region $R_{d}$. Within this region, soft labels are generated based on the feature point’s distance to the original polygon boundary, and the $L_{1}$ distance loss for the predicted threshold *T* is computed as:

(20) $$\begin{array}{c} L_{t}=\frac{1}{|R_{d}|}\sum_{\left(i,j\right)\in R_{d}}|T_{i,j}-{\hat{G}}_{d(i,j)}| \end{array}$$

where ${\hat{G}}_{d}$ is the threshold label generated based on distance transformation. By computing the loss only within the boundary region $R_{d}$, this term guides the network to adaptively adjust the binarization threshold at the edges of the digital display, thereby achieving fine-grained segmentation.

### 3.3. Reading Recognition

After obtaining precise text region segmentation results in the previous stage, the final objective of CFCP is to transform pixel-level visual features into recorded industrial numerical values. Considering the dual constraints of real-time requirements and computational resources for edge deployment in industrial monitoring scenarios, this study adopts the lightweight sequence recognition network improved by Cui et al. [43] based on SVTR [44]. This network takes the Region of Interest (RoI) extracted and corrected during the detection stage as input and aims to achieve high-accuracy sequence transcription under low computational cost. In terms of architectural design, the network employs MobileNetV1 as the feature extraction backbone and intentionally adjusts the final downsampling stride to preserve spatial resolution in the vertical direction, thereby preventing the loss of morphological information of digital characters in deeper features. Simultaneously, to address the limited receptive field of convolutional networks when processing tightly arranged characters, an SVTR block [44] is embedded after the backbone to capture global contextual dependencies of the sequence using a self-attention mechanism.

Following the training strategy of the original method, we employ a multi-head joint supervision mechanism during the training phase, utilizing both the Connectionist Temporal Classification (CTC) [45] and the Show, Attend, and Read (SAR) [46] branches to jointly constrain feature learning. Specifically, the CTC branch maximizes the conditional probability of the ground truth label sequence *Y* given the input features *X*, without requiring explicit character alignment. Its loss function $L_{ctc}$ is defined as the negative log-likelihood of the ground truth path:

(21) $$\begin{array}{c} L_{ctc}=-\log P\left(Y|X\right) \end{array}$$

Meanwhile, the SAR branch captures semantic dependencies between characters via an attention mechanism. At each time step *t*, it computes the cross-entropy loss Lsar based on the historical predictions y<t and the visual features *V*:

(22) $$\begin{array}{c} L_{sar}=-\sum_{t=1}^{T}\log P\left(y_{t}|y_{<t},V\right) \end{array}$$

To guide the network in generating more discriminative feature representations, the final total loss function $L_{rec}$ is formulated as a weighted sum of the two:

(23) $$\begin{array}{c} L_{rec}=\lambda_{ctc}L_{ctc}+\lambda_{sar}L_{sar} \end{array}$$

where $\lambda_{ctc}$ and $\lambda_{sar}$ are balancing coefficients. In the practical deployment phase of CFCP, to significantly reduce inference latency without sacrificing accuracy, we retain only the computationally efficient CTC branch for fast decoding. The final output discrete character sequence undergoes intra-line clustering and horizontal ordering based on geometric coordinates, and potential false positives from background noise are filtered out via a confidence threshold, thereby synthesizing the final meter reading.

## 4. Experiments

In this chapter, we provide a comprehensive description of the experimental details, covering the dataset construction process, parameter settings of CFCP, and the evaluation metrics used to quantify model performance. To validate the effectiveness of the proposed algorithm and the rationality of the overall architecture, we design a series of comparative experiments and ablation studies, and conduct quantitative analysis on the experimental results.

### 4.1. Dataset

#### 4.1.1. Data Collection

To validate the effectiveness of the proposed method in practical applications, we constructed a dedicated industrial instrument dataset comprising 2221 high-resolution images. All images were captured using a high-resolution industrial camera (HIKROBOT MV CE013 80UC with 1.3 megapixels, manufactured by Hangzhou Hikrobot Technology Co., Ltd. in Hangzhou, China, and procured in China) within a real instrument inspection laboratory environment, ensuring the authenticity and imaging quality of the data source. The subjects covered various types of real industrial instruments, including digital display meters with different measurement ranges and appearances. To ensure data diversity and simulate realistic inspection viewpoints, we deliberately avoided intervening in the ambient lighting during acquisition. Instead, images were obtained by varying the shooting angles and distances. Although captured in a controlled laboratory setting, most industrial instruments are equipped with glass covers or metal casings. Consequently, as the shooting angle changed, specular reflections, shadow occlusions, and low-brightness areas due to exposure variations naturally appeared in the images. Simultaneously, all images were manually and accurately annotated using the LabelMe tool v4.6.0.

#### 4.1.2. Data Augmentation

**Figure 6:**
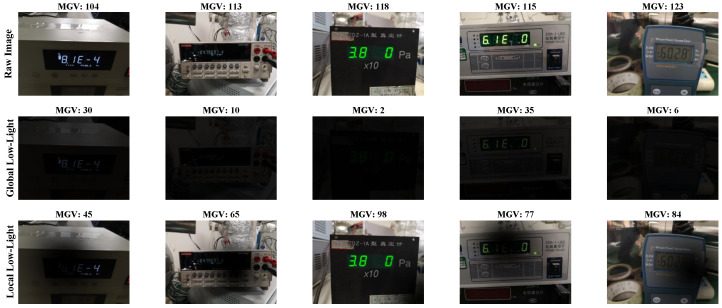
Examples of partial original samples and their augmented counterparts. A lower Mean Gray Value (MGV), closer to 0, indicates lower image brightness.

**Figure 7:**
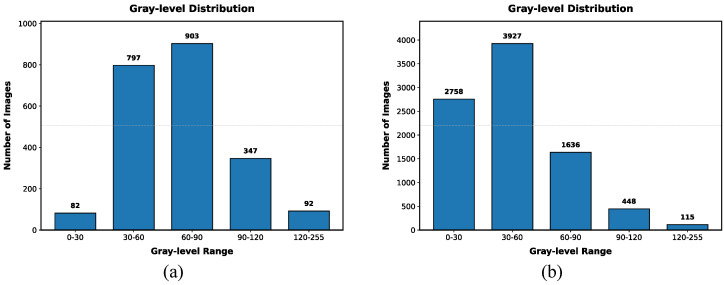
Distribution of low-light image counts across different gray-level intervals. (**a**) The quantity distribution of images across different gray levels in the constructed original dataset; (**b**) The quantity distribution of images across different gray levels in the augmented dataset.

Considering that the scale of the originally collected dataset is limited, and to enable the AICM to learn robust physical illumination recovery capabilities, we designed an illumination enhancement strategy. Different from conventional geometric transformations, this study focuses solely on simulating extreme and non-uniform low-light environments, generating synthetic data via code to augment the dataset. The specific enhancement methods include two modes: global linear darkening and local shadow simulation. To simulate globally extreme darkness such as at night or with lights off, we apply linear multiplication to attenuate the overall image brightness. Assuming the original pixel value is I(x,y) and the enhanced pixel value is $I^{\prime }\left(x,y\right)$, the global linear darkening is computed as follows:

(24) $$\begin{array}{c} I^{\prime }\left(x,y\right)=I\left(x,y\right)\times \left(1-\alpha \right) \end{array}$$

where α is the light attenuation coefficient. To simulate extreme environments, α is randomly sampled within the range [0.8,0.95], meaning the image retains only 5% to 20% of its original brightness. Local shadows are simulated to represent the partial dark areas commonly caused by equipment occlusion or point light source attenuation in industrial settings. We employ a two-dimensional Gaussian distribution to generate a random shadow mask *M*. The mask center $\left(c_{x},c_{y}\right)$ and radius *r* are randomly generated within the image boundaries. The pixel values within the shadow region undergo non-uniform attenuation based on Gaussian kernel weights, thereby creating realistic localized shading effects in the image. For each original image, we generate one globally low-light image and two locally shadowed images, expanding the dataset to four times its original size. Consequently, the augmented dataset comprises a total of 8884 images. These are split into a training set of 5276 images, a validation set of 1724 images, and a test set of 1884 images for subsequent experimental evaluation. We adopt a random split strategy, where different sets may contain the same types of meters, but the readings are distinct. Figure 6 visually presents a comparison between the original data and samples enhanced by the two aforementioned strategies. It can be observed that the enhanced images significantly increase the difficulty of recognition. Furthermore, Figure 7 contrasts the grayscale value distributions of images before and after enhancement. Statistical analysis indicates that the distribution of the enhanced data shifts markedly to the left (i.e., pixel values tend toward 0), thereby further enriching the sample characteristics of non-uniform low-light environments.

### 4.2. Experimental Settings

#### 4.2.1. Evaluation Metrics

Given that CFCP comprises two key stages, namely reading region detection and sequence recognition, different evaluation metrics are employed to more precisely quantify the performance of each stage. For assessing the performance of the first stage, Reading Region Detection, we utilize Precision (P), Recall (R), and F1-score. Precision reflects the proportion of predicted instrument regions that are genuinely instruments; Recall indicates the proportion of all actual instrument targets that are successfully located; and the F1-score provides a comprehensive evaluation of detection robustness. The formulas for calculating these metrics are defined as follows:

(25) $$\begin{array}{c} P=\frac{TP}{TP+FP} \end{array}$$

(26) $$\begin{array}{c} R=\frac{TP}{TP+FN} \end{array}$$

(27) $$\begin{array}{c} F1=\frac{2\times P\times R}{P+R} \end{array}$$

In this definition, the Intersection over Union (IoU) threshold is set to 0.5. True Positives (TP) refer to correctly detected samples where the IoU between the predicted bounding box and the ground truth box exceeds 0.5. False Positives (FP) represent false detections, which may correspond to background areas or samples with significant localization errors. False Negatives (FN) denote actual instrument targets that are missed by the detection model. Meanwhile, Average Precision (AP) represents the integral area under the Precision Recall curve. It comprehensively reflects the detection performance at various thresholds:

(28) $$\begin{array}{c} AP=\int_{0}^{1}P\left(R\right)dR \end{array}$$

Then the mean Average Precision (mAP) can be calculated using the following formula:

(29) $$\begin{array}{c} mAP=\frac{1}{N}\sum_{i=1}^{N}AP_{i} \end{array}$$

Furthermore, to evaluate the performance of the second stage, namely reading recognition, we adopt reading recognition Accuracy (Acc) as the core metric. Acc is defined as the proportion of test samples for which the digital sequence output by the system exactly matches the ground truth label. The calculation formula is as follows:

(30) $$\begin{array}{c} Acc=\frac{N_{correct}}{N_{total}} \end{array}$$

where $N_{correct}$ denotes the number of images for which the recognition result perfectly matches the ground truth character sequence, and $N_{total}$ represents the total number of images in the test set. A sample is only considered correct if the detected reading region is accurate and all digital characters are transcribed correctly.

#### 4.2.2. Training Details

All model training and inference in this study were implemented under the Ubuntu 22.04 LTS operating system using the PyTorch 2.5.0 deep learning framework, with hardware acceleration performed on an NVIDIA GeForce RTX 3090Ti GPU. During the data preprocessing stage, the input images for the detection network were uniformly resized to a resolution of 640×640. CFCP is designed as a two-stage detection-recognition model, where detection and recognition are trained respectively. For the YOLOv10 model used in the coarse detection stage, we fine-tuned based on the pre-trained YOLOv10-N [20]. The AdamW optimizer [47] was employed to optimize network parameters, with momentum parameters set to $\beta_{1}=0.9$, $\beta_{2}=0.999$, and a weight decay coefficient of $1\times 10^{-4}$. Regarding the training strategy, the initial learning rate was set to $1\times 10^{-4}$ and dynamically decayed over 500 epochs using a cosine annealing schedule, with a batch size of 64. According to the joint loss function defined in Section 3.2.4, the balancing coefficients α and β for the detection task were fixed at 1.0 and 10.0, respectively, while the weight λ for the illumination physical constraint loss was set to 0.3 to balance the gradient contributions from illumination enhancement and feature learning. Additionally, the binarization threshold was set to 0.3 in the reading stage. To ensure strict reproducibility of the experiments, the random seed for all training and testing processes was uniformly fixed to 400. The total training time for 500 epochs was approximately 8 h. During inference, a rigorous latency measurement protocol was adopted. The hardware precision mode was set to single-precision floating-point (FP32), and the input image batch size was strictly set to 1. The final reported per-sample inference latency covers the complete processing pipeline, including data loading, meter reading region localization, and final digit sequence recognition.

### 4.3. Comparisons with the State-of-the-Arts

To comprehensively evaluate the proposed CFCP on the Digital Meter Reading Recognition (DMRR) task, we compare it with two mainstream categories of methods, including Two-Stage Detection-Recognition Methods and End-to-End Sequence Recognition Methods. Among the two-stage methods, we select Faster R-CNN [48] as a representative high-precision detector, which achieves fine-grained localization through its Region Proposal Network (RPN). We also include EfficientDet-D2 [49] to assess the computational efficiency advantage of its Weighted Bidirectional Feature Pyramid Network (BiFPN), and the YOLO-CPDM+EERRM [40] method specifically designed for DMRR tasks as a benchmark for industrial-grade real-time detection solutions. Within the end-to-end methods, we adopt the classic CRNN [16], which integrates convolutional and recurrent neural networks for variable-length sequence recognition and serves as a widely used industrial benchmark for sequence recognition.

The experimental results are presented in Table 1. Comparative experiments were conducted under identical hardware environments and on the proposed dataset. The results demonstrate that CFCP achieves an optimal balance between recognition accuracy and computational efficiency. Specifically, the two-stage detector Faster R-CNN attains the highest detection accuracy (96.5%) and mAP50, owing to its refined region proposal mechanism. However, due to the lack of a dedicated illumination correction strategy, its final reading accuracy is only 92.3%. Moreover, its parameter count reaches 28.4 M and the inference latency is 46.7 ms, severely limiting its deployment feasibility on resource-constrained edge devices. In contrast, EfficientDet-D2 and YOLO-CPDM+EERRM, while improving inference speed through architectural optimizations, exhibit insufficient robustness in extreme low-light conditions, leading to relatively lower final recognition accuracy. On the other hand, the end-to-end CRNN, based on sequence recognition, although lightweight in structure, shows weaker overall performance due to its lack of a mechanism to counteract non-uniform low-light interference. Benefiting from the synergistic enhancement mechanism of AICM and IFPM, as well as the lightweight recognition backbone network, CFCP not only achieves the highest detection F1-score of 97.1% but also attains the best recognition accuracy of 93.4%. More importantly, CFCP simultaneously maintains the smallest model parameter count and the fastest inference speed, with its inference efficiency being approximately 2.7 times that of Faster R-CNN. These experimental results indicate that CFCP significantly reduces computational overhead while maintaining high recognition accuracy, making it suitable for deployment in real metrology and industrial scenarios to accomplish DMRR tasks.

To further evaluate the cross-environment generalization capability of CFCP, we conducted external validation on the Digital Meter dataset (https://aistudio.baidu.com/datasetdetail/215211) (accessed on 15 March 2026) proposed by Baidu PaddlePaddle [43]. Two evaluation protocols were designed, including direct detection-recognition and retraining. Table 2 reports the direct inference performance of all models without fine-tuning. When confronted with unseen data distributions and scene illumination, all compared models exhibited performance degradation, with recognition accuracy dropping significantly. This indicates that traditional methods are prone to overfitting specific training sets. In contrast, CFCP demonstrated strong robustness, maintaining a stable recognition accuracy of 83.2%. This convincingly demonstrates that AICM and IFPM successfully decouple environmental interference from digit character structures, enabling CFCP to learn illumination-invariant visual features. Subsequently, we fully retrained all frameworks on this dataset, with the results presented in Table 3. Due to the relatively lower scene complexity of this public dataset, the overall performance of all models was significantly improved. Even in this relatively simple recognition task, CFCP maintained a robust lead.

**Figure 8:**
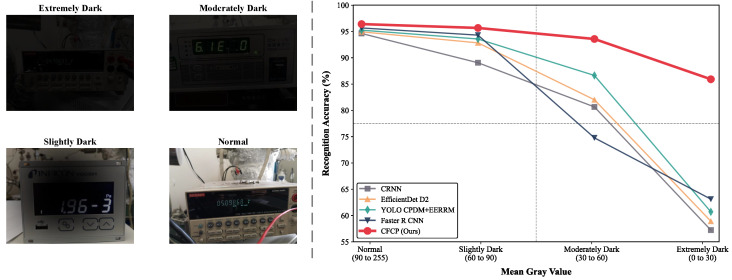
Robustness evaluation of CFCP under different brightness levels. (**Left**) representative images of four physical brightness levels categorized by average grayscale value. (**Right**) recognition accuracy degradation curves of different methods.

To objectively evaluate the robustness of CFCP under extreme illumination conditions, we strictly divided the test images into four physical brightness levels according to their average grayscale values. Figure 8 presents examples of images at each brightness level along with the corresponding recognition accuracy degradation curves. Under normal and slightly dark conditions, all compared baselines exhibited high recognition accuracy. However, when the ambient luminance drastically dropped to the extremely dark range, the performance of conventional models experienced a severe catastrophic degradation. In contrast, CFCP demonstrated a gradual performance decline and maintained a relatively high recognition rate even in extremely dark environments. These experimental results further validate the effectiveness of our AICM and IFPM, enabling CFCP to correct illumination and extract illumination-invariant digit character features under severely adverse low-light conditions.

### 4.4. Ablation Experiments

In this section, we conduct ablation studies to validate the rationale of different modules. To comprehensively evaluate the effectiveness of CFCP for digital meter reading recognition under non-uniform low-light conditions, we compare the performance using three metrics on the constructed dataset: Precision, Recall, and F1-score.

#### 4.4.1. Effectiveness of AICM

**Figure 9:**
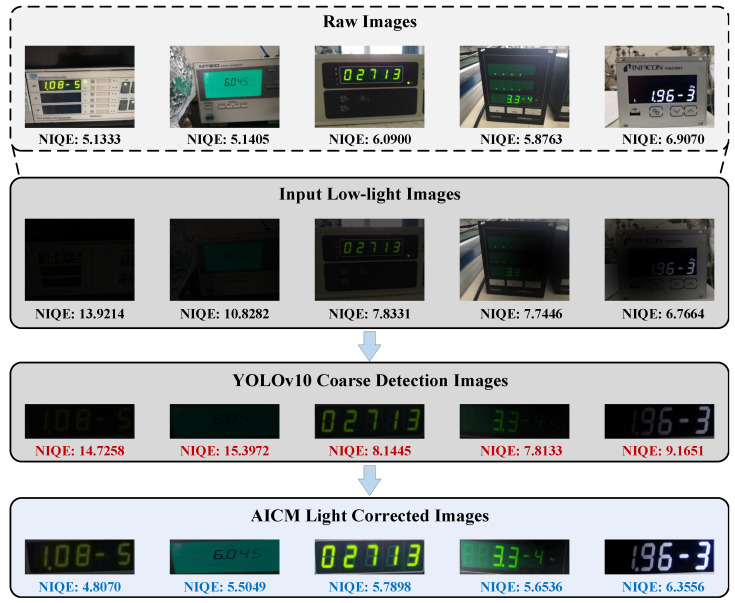
Illumination correction effect of AICM on images after coarse detection and quantitative NIQE evaluation. Natural Image Quality Evaluator (NIQE) is a no reference objective metric for image quality assessment. A lower NIQE score strictly indicates less structural distortion and higher visual naturalness.

The Adaptive Illumination Correction Module (AICM) is a core component designed to address the challenge of reading difficulty for digital display instruments under non-uniform low-light conditions. To validate its effectiveness, we compare the Baseline model (CFCP with AICM and IFPM removed) with the model incorporating AICM (Baseline+AICM) in our constructed dataset. The specific results are listed in the first two rows of Table 4. The experimental data indicate that the Baseline model lacks a dedicated illumination processing mechanism and therefore struggles to adapt to detecting digital regions against dark backgrounds. Its F1-score reaches only 69.7%, and the final recognition accuracy falls to 60.3%. After integrating the AICM, the overall performance of the model improves substantially. Detection Precision increases from 69.3% to 89.5%, and recognition accuracy increases to 85.0%. This significant performance improvement fully demonstrates the necessity of AICM for CFCP. Furthermore, it corroborates that for CFCP, detection results and recognition results exhibit a strong positive correlation. Specifically, after restoring visual information through AICM, more precise detection and localization directly contribute to more accurate reading recognition. As visualized in Figure 9, when processing low-light reading region images obtained via coarse localization, AICM effectively and rationally enhances illumination through physics-model-constrained illumination decomposition and reconstruction. This process makes character features originally obscured in shadows or low-exposure areas clearly visible, thereby providing a reliable foundation for subsequent key feature extraction.

To objectively quantify the improvement in overall image quality after processing low-light images with AICM, we introduce the Natural Image Quality Evaluator (NIQE). As illustrated in Figure 9, we track the quality score variations of images across different processing stages. A lower NIQE score indicates higher visual naturalness and fewer distortion artifacts. The cropped low-light target images from initial detection exhibit severe luminance degradation and noise, resulting in extremely high NIQE scores. After adaptive illumination correction by AICM, the NIQE scores of all samples decrease significantly. More importantly, the NIQE scores of images corrected by AICM approach or even fall below those of the original images unaffected by low-light interference. This dual comparison, both visual and quantitative, conclusively demonstrates that the AICM module effectively preserves the natural structural integrity of digital meter sequences while restoring physical illumination.

#### 4.4.2. Effectiveness of IFPM

The Illumination-invariant Feature Perception Module (IFPM) is a key component for enhancing the robustness of feature extraction in CFCP under non-uniform low-light interference. As shown in Table 4, comparing the Baseline model with the model incorporating IFPM (Baseline+IFPM) demonstrates that even without AICM for explicit illumination correction, IFPM still increases Precision from 69.3% to 77.2% and raises the F1-score to 78.5%. These results indicate that IFPM can effectively perceive and extract intrinsic structural features independent of illumination intensity from the degraded image. By focusing on the geometric form of digits rather than their surface pixels, IFPM successfully filters out noise caused by specular reflections or local shadows, thereby reducing the false positive rate. Furthermore, when AICM and IFPM work together, the F1-score reaches 97.1%, and the final recognition accuracy improves to 93.2%. This significant improvement validates the complementary roles of the two modules in physical space and feature space. While AICM effectively restores the visual visibility of images, its brightness enhancement process inevitably amplifies background noise. At this stage, IFPM plays a crucial role at the feature level. By extracting highly discriminative structural features, it effectively filters out the interference noise highlighted by AICM. The synergistic action of both modules jointly ensures accurate meter reading for industrial digital instruments in non-uniform low-light scenarios.

**Figure 10:**
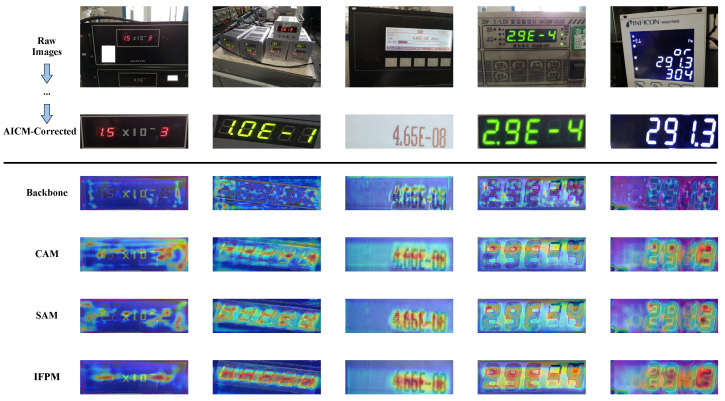
Visual ablation comparison of feature attention in the IFPM module. From top to bottom: original image, AICM-corrected image, feature heatmaps from the backbone only (Backbone), channel attention only (CAM), spatial attention only (SAM), and the complete IFPM (CAM+SAM). Red regions represent high-response activation areas. For clarity, the visualization from the original image to AICM correction is omitted.

To further validate the design rationality of IFPM, we conducted a visual ablation analysis on its internal CAM and SAM components. The experimental results shown in Figure 10 indicate that applying CAM or SAM individually can enhance feature responses to some extent. However, when dealing with the background noise amplified by AICM, these individual modules still exhibit scattered attention or incomplete noise suppression. In contrast, the complete IFPM successfully concentrates the high-response areas of the network precisely onto the reading region through the synergistic calibration of channel filtering and spatial localization. This demonstrates the necessity of combining both modules for effective noise suppression and extraction of illumination-invariant features.

#### 4.4.3. Effectiveness of Recognition Network

To validate the adaptability of the improved lightweight recognition network used in this study for industrial edge deployment, we compare this recognition component within CFCP against CRNN [16], Rosetta [50], and the original SVTR-Base [44]. The results are presented in Table 5. Experimental data indicate that while the classic CRNN model holds certain advantages in terms of parameter count and inference time, its RNN-based sequence modeling capability is limited when processing tightly arranged digital characters. Its Accuracy reaches only 91.4%, which is insufficient to meet the requirements of high-precision monitoring. In comparison, the lightweight network integrated into CFCP enhances the ability to capture global character dependencies by incorporating the SVTR block. While maintaining low parameter count and low inference latency, it significantly improves Accuracy to 93.2%. On the other hand, although SVTR-Base achieves the highest recognition accuracy with its complex deep architecture, its parameter count reaches 24.6 M, and its single-sample inference time is relatively long. Benefiting from the effective combination of the MobileNetV1 [51] backbone and the CTC decoding strategy, the recognition model employed in CFCP reduces the parameter count by approximately 56% compared to SVTR-Base while incurring only a 1.9% loss in Accuracy, and achieves faster inference response.

**Figure 11:**
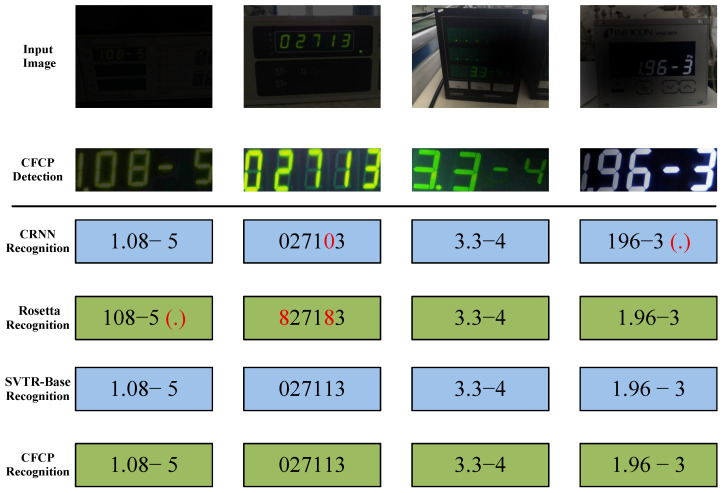
Recognition results of CFCP using different recognition networks, with the input and the CFCP detection process kept identical. Red text indicates recognition errors, and characters within () denote missed characters. Note that both SVTR-Base and CFCP support space recognition.

To visually validate the effectiveness of the recognition model, Figure 11 presents a qualitative comparison of results from different recognition networks given the same detection input. It can be observed that when dealing with decimal points commonly present in industrial digital instruments and prominent transistor contours, both CRNN and Rosetta exhibit missed detections and misidentifications due to confusing these transistor contours with the reading digits. In contrast, the recognition network adopted by CFCP, leveraging its ability to perceive global context, accurately reconstructs all character details, including spacing and decimal points. This further demonstrates the robustness of this network in handling highly similar characters and subtle semantic features. The experimental results presented above indicate that CFCP effectively achieves a balance between recognition accuracy and computational cost, making it more suitable for industrial digital meter reading tasks that demand both real-time performance and high accuracy.

#### 4.4.4. Effectiveness of Parameter λ

**Figure 12:**
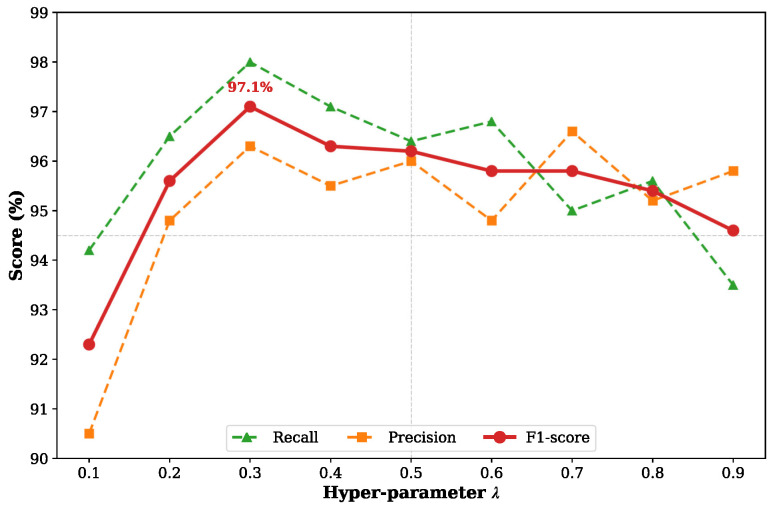
Influence of different λ values in Equation (16) on the fine detection results. Corresponding to physical constraints of varying strengths, variations in λ affect the model’s final Precision, Recall, and F1-score for fine detection of the reading area.

In CFCP, the AICM utilizes a physically constrained loss to guide the reconstruction of the illumination component. The hyperparameter λ (Equation (16)) directly controls the strength of this constraint, thereby governing the trade-off between smoothness in illumination correction and detail preservation. As shown in Figure 12, experiments conducted over the range λ∈[0.1,0.9] reveal that when λ is small, insufficient physical prior constraints inevitably lead the AICM to amplify certain background noise while enhancing brightness. This results in lower Precision and consequently restricts overall performance. As λ increases to 0.3, the F1-score reaches its peak at 97.1%. At this point, the model attains its optimal balance. With moderate physical constraints, the AICM effectively enhances illumination, raising Precision to 96.3%, while fully preserving the edge details of digital characters and maintaining a high Recall. However, when λ increases further, excessive regularization forces the illumination component to become overly smooth, causing some digital character details to be obscured by overexposure, which leads to a decline in overall detection performance.

### 4.5. Convergence and Overfitting Analysis

**Figure 13:**
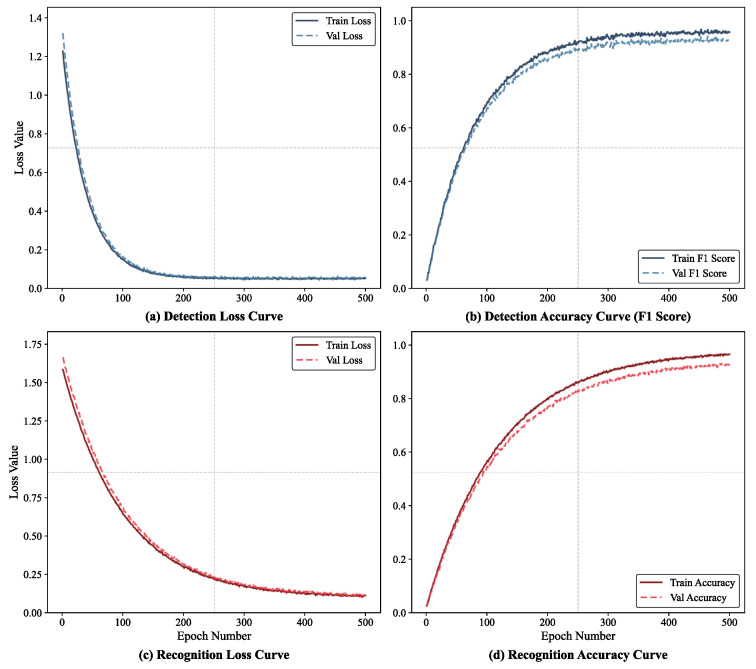
Training and validation convergence curves of CFCP over 500 training epochs. (**a**,**b**) illustrate the loss and F1-score during the detection stage, respectively. (**c**,**d**) present the loss and accuracy during the recognition stage, respectively.

To comprehensively evaluate the convergence behavior of CFCP and verify its generalization capability, we comprehensively recorded the training process of both the detection and recognition stages. Figure 13 illustrates the loss and accuracy trends over 500 training epochs. Figure 13a,b present the metric variations during the detection stage. The detection network rapidly learned coarse geometric localization features in the early phase. Its training and validation losses declined rapidly during the initial stage and subsequently plateaued, while the F1-score exhibited a smooth upward trend. As shown in Figure 13c,d, the digit sequence recognition task requires extracting fine-grained semantic features, resulting in a relatively smoother and longer convergence process. The recognition loss and accuracy demonstrated a stable optimization trend throughout the entire 500 epochs. In both independent training stages, the validation curves consistently closely followed the training curves. No rebound phenomenon was observed in the validation loss during the later training phase. The above experimental results fully demonstrate that CFCP updates network weights healthily and does not exhibit overfitting to the training data.

### 4.6. Failure Cases

**Figure 14:**
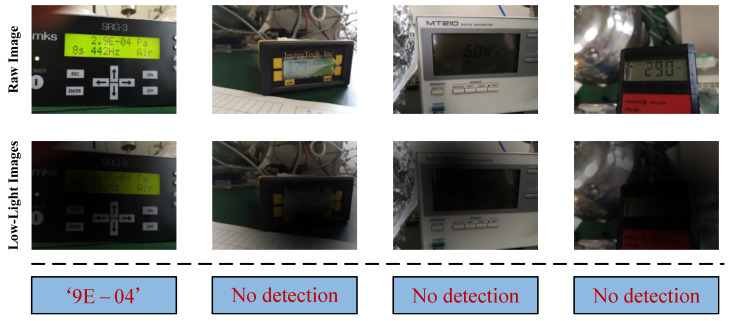
Typical failure cases of CFCP under non-uniform low-light environments. The leftmost column of images shows incomplete recognition results, while the subsequent three columns illustrate complete missed detections.

This section presents an objective analysis of the failure cases of CFCP under non-uniform low-light conditions. Figure 14 illustrates representative samples where the model produces erroneous predictions, with these failure cases primarily revealing two types of limitations inherent in the framework. The first limitation manifests as incomplete recognition caused by large-field backgrounds and extremely small reading targets. As shown in the first column of images on the left, the reading region occupies a minimal pixel proportion in the global image. Coupled with the masking effect of non-uniform low-light illumination, this makes it challenging for the coarse detection module of CFCP to accurately delineate the boundaries of tiny reading areas. Consequently, the generated bounding boxes truncate the leading digit characters, resulting in the model ultimately outputting incomplete reading sequences. The second limitation stems from complete missed detections induced by background or local meter highlights and specular reflections. As illustrated in the subsequent three columns of images, extreme low-light conditions interact synergistically with meter screen reflections or background highlighted regions. While AICM restores image brightness, it simultaneously amplifies these highlighted areas, causing the model attention to become excessively concentrated on such regions and leading to the neglect of critical digit stroke features. The detection network consequently fails to extract effective visual targets, and the model directly outputs null results at the detection stage. In summary, the primary limitations of the current CFCP remain concentrated in the front-end detection stage. Addressing the aforementioned issues will further enhance the accuracy guarantee of CFCP in real-world application scenarios and advance the development of two-stage DMRR methodologies.

## 5. Discussion

Despite the promising performance of CFCP in non-uniform low-light environments, several objective limitations remain. First, the current model is primarily optimized for illumination degradation. In real-world complex industrial scenarios, digital meters often suffer from extreme physical obstructions, including severe mud contamination, cracked dial glass, or intense local specular reflections. Furthermore, pure spatial perception models face severe visual ambiguity when capturing the exact moment of a screen refresh. This occurs when fading residual digits and new digits overlap. The robustness of the proposed framework remains limited when handling such physical damage that disrupts the digital topological structure. Second, although the two-stage decoupled architecture ensures system stability, it inevitably introduces the risk of cascaded error propagation. Once the front-end detector suffers from severe misdetection or false detection, the subsequent recognition module is unable to rectify the error.

Future work will focus on overcoming the aforementioned limitations. We will dedicate efforts to collecting and open-sourcing a large-scale industrial dataset containing multiple complex physical degradations. Specifically, we will directionally collect extreme data that display ghosting features. This will force the model to learn subtle contrast differences between active characters and fading residuals. To further explore model quantization and deployment on resource-constrained edge devices, we will investigate lightweight and acceleration techniques for practical engineering applications. Furthermore, extending the perception dimension of CFCP to adapt to a wider range of industrial metrology equipment, such as analog pointer-type meters, will also constitute an important research direction in our next steps.

## 6. Conclusions

This paper presents a Coarse-to-Fine Cascade Perception framework (CFCP) for digital meter reading recognition in industrial environments characterized by non-uniform low-light conditions. The core contribution of this study lies in addressing the challenge of simultaneously performing illumination restoration and noise suppression, which is often neglected by existing methods. Specifically, the Retinex-based Adaptive Illumination Correction Module (AICM) achieves the decoupling and correction of illumination components at the pixel level, while the Illumination-invariant Feature Perception Module (IFPM) effectively suppresses background noise at the feature level. This synergistic mechanism of pixel-level illumination correction and feature-level noise suppression significantly enhances the adaptability of CFCP to non-uniform low-light environments. Experimental results on the self-constructed industrial digital meters dataset demonstrate that CFCP achieves a recognition accuracy of 93.2% with a single-sample inference latency of only 17.1 ms, satisfying the industrial deployment requirements for both real-time performance and lightweight design.

## Figures and Tables

**Table 1:** Comparison Results of CFCP with Different Methods. Params and Time denote the number of parameters and the single-sample inference latency of each method, respectively. All models are implemented under the same hardware environment. To ensure robustness against experimental randomness, the recognition stage is trained five times with different random seeds, reporting the mean and standard deviation of recognition accuracy (Acc). The best results are marked in **bold**.

Methods	P	R	F1	mAP50	Acc	Params (M)	Time (ms)
CRNN	-	-	-	-	91.8 ± 0.3	8.3	20.3
EfficientDet-D2	94.1	95.5	94.8	95.3	91.5 ± 0.2	8.1	18.4
Faster R-CNN	**96.5**	95.8	96.1	96.4	92.3 ± 0.3	28.4	46.7
YOLO-CPDM+EERRM	95.2	96.8	96.0	96.8	91.6 ± 0.4	12.8	30.2
**CFCP**	96.3	**98.0**	**97.1**	**98.4**	**93.4 ± 0.1**	**7.9**	**17.1**

**Table 2:** Evaluation results for detection and recognition on the Digital Meter dataset using the non-fine-tuned model. The best results are marked in **bold**.

Methods	P	R	F1	mAP50	Acc
CRNN	-	-	-	-	78.2 ± 0.5
EfficientDet-D2	84.1	85.2	84.6	86.3	74.1 ± 0.4
Faster R-CNN	88.5	87.2	87.8	87.1	80.3 ± 0.5
YOLO-CPDM+EERRM	86.4	88.1	87.2	85.3	81.9 ± 0.3
**CFCP**	92.4	**93.1**	**92.7**	**91.7**	**83.2 ± 0.2**

**Table 3:** Evaluation results of the model retrained on the Digital Meter dataset. The best results are marked in **bold**.

Methods	P	R	F1	mAP50	Acc
CRNN	-	-	-	-	93.5±0.3
EfficientDet-D2	95.4	96.2	95.8	96.3	93.8±0.2
Faster R-CNN	97.8	96.6	97.2	97.5	94.5±0.1
YOLO-CPDM+EERRM	96.5	97.5	97	98.6	94.1±0.1
**CFCP**	**97.5**	**98.8**	**98.1**	**99.1**	**96.2 ± 0.1**

**Table 4:** Ablation study of different components of CFCP on the self-constructed dataset. The method with AICM and IFPM removed from CFCP serves as the Baseline, and the best results are highlighted in **bold**.

Model	AICM	IFPM	Precision	Recall	F1-Scores	Acc
Baseline	×	×	69.3	70.2	69.7	60.3
Baseline+AICM	✓	×	89.5	88.7	89.1	85.0
Baseline +IFPM	×	✓	77.2	79.8	78.5	65.4
**CFCP**	✓	✓	**96.3**	**98.0**	**97.1**	**93.2**

**Table 5:** Ablation study of different recognition models for CFCP on the self-constructed dataset. To ensure experimental fairness, the detection stage is kept consistent. After completing the reading area detection, the detection results are respectively fed into different recognition models for meter reading recognition. The best results are marked in **bold**.

Recognition Model	Acc	Params	Time
CRNN	91.2	8.3 M	20.3 ms
Rosetta	90.6	44.3 M	67.5 ms
SVTR-Base	**95.1**	24.6 M	43.8 ms
**CFCP**	93.2	**4.9 M**	**11.5 ms**

## Data Availability

The data supporting the findings of this study have been included in this paper. Due to privacy policies, the data presented in this study are available on request from the corresponding author.

## Abbreviations

The following abbreviations are used in this manuscript:

DMRR	Digital Meter Reading Recognition
CFCP	Coarse-to-Fine Cascaded Perception
TLICN	Two-Level Illumination Correction Network
AICM	Adaptive Illumination Correction Module
IFPM	Illumination-invariant Feature Perception Module
SHN	Stacked Hourglass Network
LLIE	Low-Light Image Enhancement
HE	Histogram Equalization
RoI	regions of interest
GAP	Global Average Pooling
CAM	Channel Attention Module
SAM	Spatial Attention Module
MLP	Multi-Layer Perceptron
DB	Differentiable Binarization
OHEM	Online Hard Example Mining
BCE	Binary Cross-Entropy
CTC	Connectionist Temporal Classification
SAR	Show, Attend, and Read
MGV	Mean Gray Value
BiFPN	Bidirectional Feature Pyramid Network
